# Effect of Long-Term Fish Oil Supplementation on Semen Quality and Serum Testosterone Concentrations in Male Dogs

**DOI:** 10.22074/ijfs.2016.4913

**Published:** 2016-06-01

**Authors:** Analía Risso, Francisco Javier Pellegrino, Alejandro Enrique Relling, Yanina Corrada

**Affiliations:** 1 Veterinary Genetic Institute (IGEVET), Veterinary College (FCV), La Plate National University (UNLP), Buenos Aires, Argentina; 2 National Council of Research and Technology (CONICET), La Plata, Buenos Aires, Argentina

**Keywords:** Canine, Fatty Acids, Semen, Testosterone

## Abstract

**Background:**

Manipulating the dietary fatty acid (FA) content can alter FA profiles of reproductive tissues. Numerous researchers have evaluated the effect of fish oil (FO) supplementation on reproductive characteristics in domestic animals, but reliable information concerning dietary FO effects on semen quality and testosterone concentrations in dogs has not been reported. Therefore, this study evaluated the effects of dietary FO on semen quality and serum testosterone concentrations in dogs.

**Materials and Methods:**

In this cross-over experimental study, 5 male dogs consumed either a control diet or the same diet supplemented with 54 mg FO/kg metabolic body weight (BW) for 120 days. After the 120-day wash-out period, control (C) dogs received FO and FO-fed dogs consumed the control diet. In the first period, 2 dogs were allocated to the FO group and 3 to the C group. In the second period, 3 dogs were allocated to the FO group and 2 to the C group. Semen samples collected on days 0, 60, 90 and 120 were evaluated by standard methods. Day 120 semen samples were analyzed for FA profiles. Blood samples were collected on days 0, 30, 60, 90 and 120 to measure serum testosterone concentrations. Data were analyzed by analysis of variance with repeated measures using the Mixed Models procedure of SAS (version 9.0, SAS Institute Inc., Cary, NC, USA). Animals and period of time (first or second 120 days) were random variables; and treatment, time, and the treatment by time interaction were considered fixed effects.

**Results:**

FO supplementation increased the percentage of motile sperm (P=0.02), total sperm count (P<0.01), total sperm viability (P<0.01), and total morphologically normal sperm (P<0.01). Supplementation decreased the percentage of viable sperm (P=0.03) and serum testosterone concentration (P<0.01). FO supplementation also increased the percentage of arachidonic acid, eicosapentaenoic acid, (EPA) and total n-3 in semen samples (P≤0.05).

**Conclusion:**

These results are consistent with the concept that long-term FO supplementation influences semen quality and testosterone concentrations in dogs by altering semen FA profiles.

## Introduction

The fatty acid (FA) content in diets can be manipulated to alter FA profiles of reproductive tissues. Fish oils (FO) contain omega 3 FA (n-3) and are a major source of eicosapentaenoic acid (EPA, C20:5 n-3) and docosahexaenoic acid (DHA, C22:6 n-3) (1). Numerous researchers have evaluated the effect of FO supplementation on reproductive parameters in domestic animals. They reported improved sperm motility and decreased morphologically abnormal sperm in boars (2), increased fertility in turkeys (3), and attenuated seasonal declines in semen quality and increased total sperm count in rams (4). However, other studies conducted in boars showed that dietary FO had no effect on semen quality (5,6). FO supplementation in turkeys had no effect on sperm motility and sperm viability compared with the control group (7). Other studies reported changes in sperm FA profiles by FO supplementation in boars (5) and rams (4). 

Testosterone is a hormone essential for spermatogenesis and male fertility (8). This hormone acts synergistically with both luteinizing hormone and follicle stimulating hormone to increase spermatogenesis efficiency and fertility. Reports vary on the effect of FO supplementation on testosterone concentrations in different species. FO supplementation has been shown to increase testosterone concentrations in rats (9) and rams but decreased testosterone concentrations in boars (10). 

To our knowledge, there are no studies that have specifically evaluated sperm parameters and serum testosterone concentrations in dogs that received FO supplements. Therefore, the present study evaluated the effects of dietary FO that contained n-3 FA on semen quality and serum testosterone concentrations in dogs. We hypothesized that FO supplementation would improve semen quality and increase serum testosterone in dogs. 

## Materials and Methods

### Animals and treatments

The study was approved by the Institutional Animal Care and Use Committee (IACUC, Number 34-1-13) of the School of Veterinary Sciences, National University of La Plata, Buenos Aires, Argentina. 

All dogs started the study at the same time. One month before the study, the dogs consumed commercial balanced food (Table 1) and were trained for semen collection, performed by manual stimulation twice per week. We chose the study dogs according to age, body condition and a complete medical record (history, clinical visit and clinical examination). As complementary methods, the dogs underwent routine blood and chemical tests, along with semen evaluations. 

**Table 1:** Nutrient composition of commercial feed


Ingredient	Dry matter (%)

Protein	30.04
Fat	15.02
Fiber	1.72
Ash	7.83
Calcium	1.50
Phosporus	1.07
Mineral and vitamins Mix^*^	0.048


^*^; Concentration according to manufacturer information: con- tains 12.39% Vitamin E, 0.20% Vitamin K, 0.82% Vitamin B1, 0.82% Vitamin B2, 0.82% Vitamin B6, 0.0004 Vitamin B12, 0.12% Acid Folic, 0.10% Acid Nicotinic, 2.06% Calcium Pantothenate, 0.02% Biotin, 82.61% Colin, 0.01% copper, 0.01% iron, 0.02% zinc, 0.003% iodine, 0.01% manganese and 0.0002% of selenium in the mineral and vitamin nucleus.

This cross-over, controlled experimental trial stud- ied 5 healthy privately owned mixed-breed dogs, 2 to 5 years of age, that weighed 17.9 ± 3.10 kg, with body condition scores of 3 in on a 1 to 5 scale (11). Before the study, semen characteristics of all dogs were within normal limits with sperm concentration>200×10^6^/ml, sperm motility>70%, sperm pro- gressive motility>70%, sperm viability>80%, and morphologically abnormal sperm<20% (12). Dogs were randomly assigned to two groups, control (C) and FO. The C group only received a control diet for 120 days, whereas the FO group received the con- trol diet plus 54 mg FO/kg of metabolic body weight (BW0.75) for 120 days. This dose was supplemented orally within an enteric-coated capsule. The dura- tion of each period was to comprise two complete cycles of spermatogenesis (13). In the first period, 2 dogs were allocated to the FO group and 3 to the C group. In the second period, 3 dogs were allocated to the FO group and 2 to the C group. Finally, to avoid any carry-over effects, we included a 120-day wash-out period between treatments during which all dogs received the control diet. This time allowed for a complete FA wash-out as described previously by Cao et al. (14).

The dogs were kept in the owners´ homes dur- ing the study. The owners signed a written consent before the experiment and agreed to feed either the commercial balanced food provided (C) or the com- mercial balanced food with the supplement (FO). Water was allowed on an ad libitum basis. Daily rations were controlled according to the daily maintenance requirements. Maintenance energy requirements (MERs) were calculated based on the formula: 

$\mathrm{MER}={\left[130X(\mathrm{BW})\right]}^{0.75}$ (15). 

### Semen collection and evaluation

Semen was collected by manual stimulation. After collection, semen volume was determined in the spermatic fraction using a graduated tube. Sperm concentration (10 ^6^/ml) was determined using a Neubauer chamber, followed by a calculation of the total sperm count (volume×concentration). Subjective vigor, defined as the linearity and quality of spermatic movement, was evaluated with a 0 to 5 score as described previously by da Rocha et al. (16). The percentages of motile sperm and progressive motility were evaluated on a drop of semen placed between a slide and cover glass on a heated stage and visualized by light microscopy at x400 magnification in 10 fields. Sperm with normal morphology were evaluated in samples stained with Rose Bengal under optical microscopy at ×1000 (17); afterwards, we calculated the number of total morphologically normal sperm (total sperm count×percentage of sperm with normal morphology). The percentage of sperm viability was evaluated after supravital staining with eosinnigrosin; then, total sperm viability was calculated (total sperm count×percentage of sperm viability). All evaluations were performed in duplicate and we counted 200 sperm cells. 

### Testosterone measurement

We obtained blood samples by peripheral venipuncture at 0, 20, 40, 60, 80, 100 and 120 minutes, beginning at 09:00 on days 0, 30, 60, 90 and 120 of each period. This 2 hour window for each day was used to buffer the changes in serum testosterone concentrations. The average serum testosterone concentration for this 2 hour window was used in the statistical analysis. To reduce potential damage in successive blood extractions, we used different veins (cephalic, saphenous and right and left jugular). Blood was centrifuged at 1400 x g for 5 minutes and serum was harvested and stored frozen at -18°C until analysis. Testosterone was determined by electrochemiluminescence (Elecsys®, Cobas®, West Sussex, England), as previously validated in dogs by García Romero et al. (18). 

### Feed analysis

Feed was pooled and analyzed for dry matter (80ºC for 48 hours), neutral detergent fiber (NDF, Ankom200 Fiber Analyzer, ANKOM Technology, Fairport, NY), crude protein (CP, Kjeldahl N x 6.25), lipids (Ether extract, XT101 ANKOM Technology Method 2) and ash (Table 1) (19). 

### Lipid analysis of feed, fish oil and spermatic fraction

We analyzed a pool of feed and supplemental FO to evaluate the FA profile (Table 2). Semen samples for lipid analysis were collected on days 120 of each period and stored at -80ºC until analysis. 

**Table 2:** Fatty acid profile (%) of commercial feed and fish oil


Fatty acid	Commercial feed	Fish oil

Tetradecanoic (14:0)	0.8	5.8
Palmitic (16:0)	19.6	24
Palmitoleic (16:1)	2.9	10.1
Stearic (18:0)	4.7	3.4
Oleic (18:1 n-9)	25.7	22.3
Vaccenic (18:1 n-7)	-	3.3
Linoleic (18:2 n-6)	38.1	2
Gamma-linolenic (18:3 n-6)	1.4	-
Alpha-linolenic (18:3 n-3)	4.0	1.1
Eicosenoic (20:1 n-9)	0.1	2.5
Dihomo-gamma-linolenic (20:3 n-6)	0.2	-
Eicosatrienoic (20:3 n-3)	0.4	-
Arachidonic (20:4 n-6)	1.4	1
Eicosatetraenoic (20:4 n-3)	-	0.8
Eicosapentaenoic (20:5 n-3)	0.1	7.6
Docosapentaenoic (22:5 n-6)	0.2	-
Docosapentaenoic (22:5 n-3)	0.2	1.2
Docosahexaenoic (22:6 n-3)	0.2	14.9
Σ SFA	25.1	33.2
Σ MUFA	28.7	38.2
Σ PUFA	46.2	28.6
Σ n-6	41.3	3
Σ n-3	4.9	25.6


SFA; Saturated fatty acids, MUFA; Monounsaturated fatty acids, PUFA; Polyunsaturated fatty acids, n-6; Omega 6, and n-3; Omega 3.

Samples for lipid extraction were analyzed following the method described by Folch et al. (20). Once lipids were obtained, they were saponified with potassium hydroxide dissolved in ethanol for 45 minutes at 80ºC, then acidified with a 0.5 ml hydrochloric acid concentrate. The acids were esterified with boron trifluoride at 64ºC for 1.5 hours. FA composition was determined by gas liquid chromatography with a 30 mm capillary column (Omega Wax 250, Supelco, Bellefonte, PA, USA). Temperature was programmed for a linear increase of 3°C per minute from 175 to 230°C. The chromatographed peaks were identified by comparing their retention times with standards. 

### Statistical analysis

We used a cross-over design with repeated measures in time. Each individual dog was considered an experimental unit. Data were analyzed with the PROC MIXED of SAS (version 9.0, SAS Institute Inc., Cary, NC, USA). The linear mixed model included the random effect of dogs, the period of the cross-over design, the fixed effect of time (0 vs. 30 vs. 60 vs. 90 vs. 120 days), treatment (C vs. FO), and time by treatment interaction. The interaction between treatment and period of the cross-over design was also included. The slice option of SAS was used to detect the time points when significant differences in the time by treatment interaction occurred when the interaction was significant. For semen FA profiles, we used only one time point. Therefore, time effect and interaction with treatment were removed from the model. 

Data are represented as least square means (LSM) ± the standard error of the means (SEM). For the main effects (time or treatment) the alpha level of significance was set at P<0.05 and P<0.1 was considered a tendency. For the interaction of the main effect (time by treatment) P<0.1 was considered statistically significant (21,22). 

## Results

### Semen evaluation

FO supplementation increased the percentage of motile sperm (P=0.02), total sperm count (P<0.01), total sperm viability (P<0.01), and total morphologically normal sperm (P<0.01). FO supplementation decreased the percentage of sperm viability (P=0.03). There was a trend (P=0.09) for an increase in the percentage of morphologically normal sperm (Table 3). 

**Table 3:** Effect of fish oil supplementation for 120 days on semen characteristics in five male dogs in the control (C, n=5) and fish oil (FO, n=5) groups


	Day 0		Day 60		Day 90		Day 120		SEM	P value	Time	FO x time
	C	FO	C	FO	C	FO	C	FO				

Semen volume (ml)	1.42	1.40	1.32	1.74	1.68	1.70	1.50	1.90	0.32	0.367	0.509	0.842
Motile sperm (%)	88.00	90.50	90.00	93.00	88.00	93.33	89.00	92.00	3.39	0.02	0.914	0.940
Sperm progressive motility (%)	86.00	83.00	89.00	89.00	86.00	91.66	85.00	92.00	4.84	0.198	0.486	0.143
Vigor	4.5	4.3	4.5	4.4	4.5	4.4	4.5	4.4	0.10	0.178	0.879	0.900
Total sperm count (10^6^ )	399.80	439.60	404.90	1105.20	649.00	1212.00	511.40	1361.00	296.45	<.001	0.001	0.020
Total sperm viability (10 6 )	338.22	379.38	322.43	806.64	549.10	904.40	419.30	963.23	267.97	<.001	0.017	0.076
Sperm viability (%)	83.60	84.60	82.00	76.00	83.00	72.13	81.00	71.40	3.31	0.03	0.030	0.458
Total morphologically normal sperm (10 6 )	311.94	376.72	295.00	843.25	541.67	1042.56	433.80	1099.60	234.50	<.001	0.005	0.020
Morphologically normal sperm (%)	79.40	84.00	73.00	78.60	83.00	87.25	81.00	81.00	3.29	0.09	0.141	0.768

C; Receiving only the control diet for 120 days and FO; Receiving 13.8 mg of n-3 from fish oil/kg of metabolic weight for 120 days.

**Fig.1:**
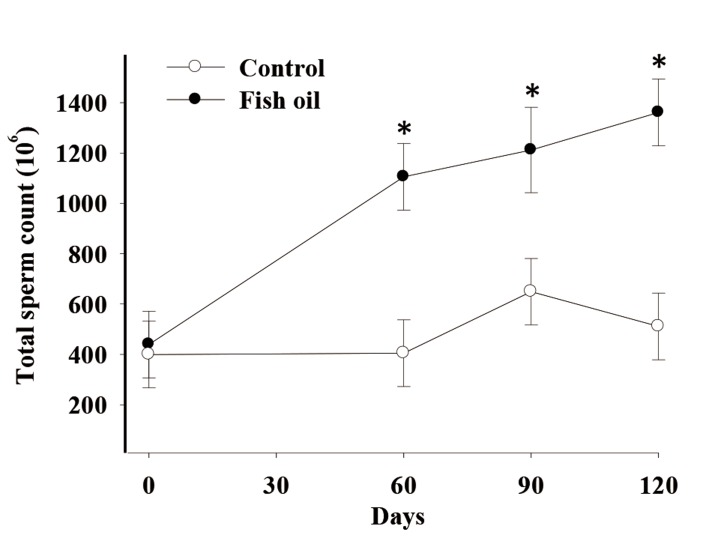
Effect of fish oil supplementation for 120 days on total sperm count in five male dogs in the control (C, n = 5) and fish oil groups (FO, n = 5). *; P=0.02, C; Receiving a control diet during 120 days and FO; Receiving 13.8 mg of n-3 from fish oil/kg of metabolic weight for 120 days.

**Fig.2:**
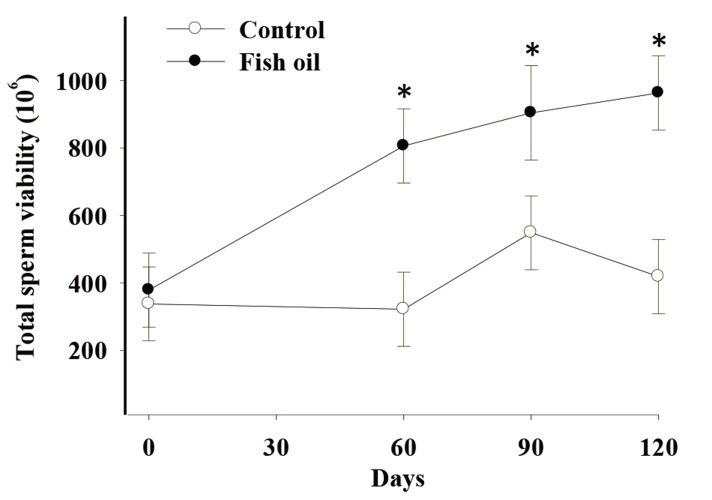
Effect of fish oil supplementation for 120 days on total sperm viability in five male dogs in the control (C, n=5) and fish oil groups (FO, n=5). * ; P=0.076, C; Receiving a control diet during 120 days and FO; Receiving 13.8 mg of n-3 from fish oil/kg of metabolic weight for 120 days.

**Fig.3:**
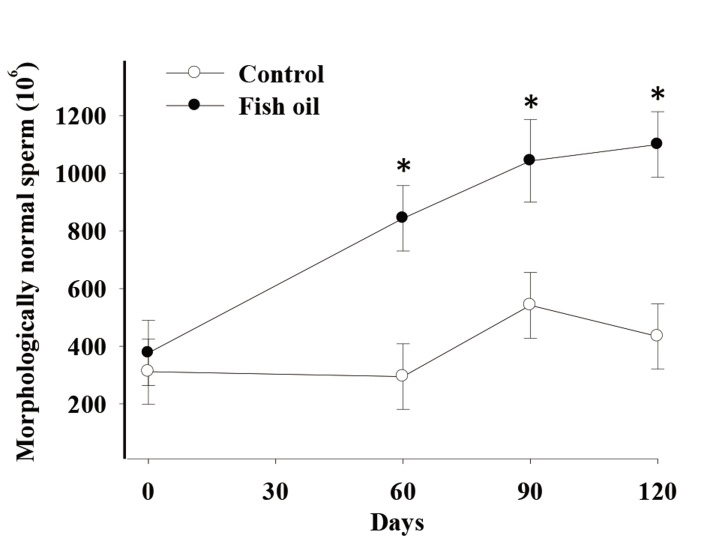
Effect of fish oil supplementation for 120 days on total morphologically normal sperm in five male dogs in the control (C, n=5) and fish oil groups (FO, n=5). ^*^; P=0.02, C; Receiving a control diet during 120 days and FO; Receiving 13.8 mg of n-3 from fish oil/kg of metabolic weight for 120 days.

There was a time by treatment effect on total sperm count (P=0.02, Fig .1), total sperm viability (P=0.076, trend, Fig .2), and total morphologically normal sperm (P=0.02, Fig .3). Conversely, we observed no effects of FO supplementation on semen volume, sperm progressive motility, and vigor (P>0.10, Table 3). 

### Testosterone concentration 

**Fig.4:**
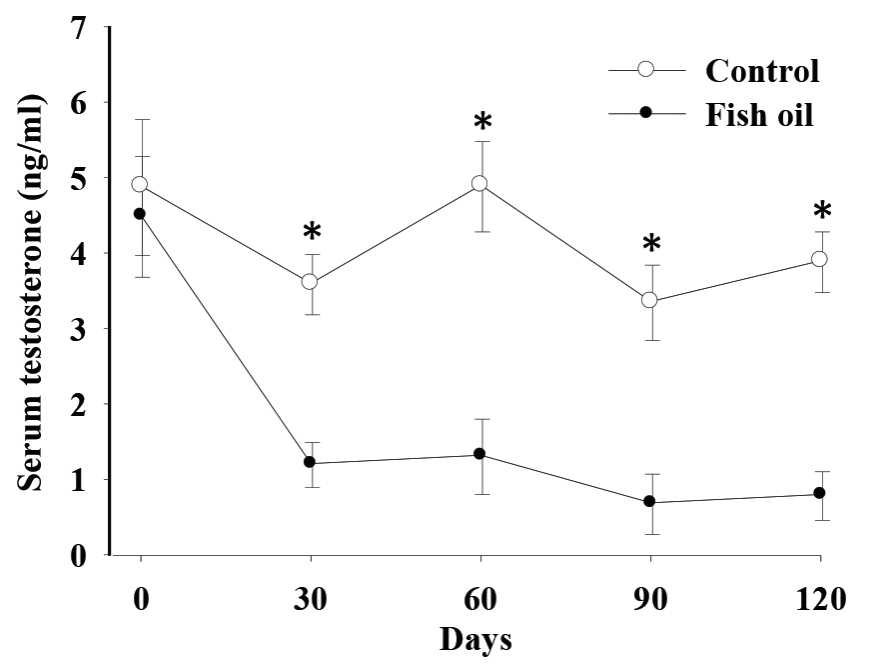
Effect of fish oil supplementation for 120 days on serum testosterone concentration in five male dogs in the control (C, n=5) and fish oil groups (FO, n=5). ^*^; P<0.05, C; Receiving a control diet during 120 days and FO; Receiving 13.8 mg of n-3 from fish oil/kg of metabolic weight for 120 days.

There was a time by treatment effect on serum testosterone concentrations (P<0.01, Fig .4). Both groups had similar serum testosterone concentrations on day 0 (4.8 ± 0.3 ng/ml). In C dogs, these concentrations did not change with time, whereas in FO dogs they decreased on day 30 (1.31 ± 0.3 ng/ml) and remained low on days 60 (1.84 ± 0.5 ng/ml), 90 (1.20 ± 0.3), and 120 (0.89 ± 0.3). 

### Fatty acid profiles of semen samples

Dogs supplemented with FO had greater semen FA concentration of arachidonic acid (AA, AA 20:4 n-6, P=0.01), EPA (20:5 n-3, P=0.02) and total n-3 (P=0.05, Table 4). Despite the increase of arachidonic acid (AA 20:4 n-6) in FO dogs, the ratio n-6/n-3 tended (P=0.09) to be smaller compared with C dogs. FO dogs tended to have higher DHA (22:6 n-3, P=0.09) and palmitoleic acid (16:1 n-7, P=0.07) but lower bosseopentaenoic acid (20:5 n-6, P=0.06) levels than C dogs. 

**Table 4:** Fatty acid composition (LSM ± SEM) of semen samples from five male dogs in the control (C, n=5) and fish oil (FO, n=5) groups at the end (120 days) of the experiment


Fatty acid	Day 120			
	C	FO	SEM	P value

Tetradecanoic (14:0)	1.55	1.81	0.11	0.155
Palmitic (16:0)	32.80	31.27	0.67	0.15
Palmitoleic (16:1 n-7)	1.29	2.00	0.23	0.07
Stearic (18:0)	13.85	13.95	0.61	0.90
Oleic (18:1 n-9)	7.34	7.92	0.90	0.66
Vaccenic (18:1 n-7)	3.42	3.39	0.32	0.93
Linoleic (18:2 n-6)	3.95	5.23	0.64	0.21
Eicosenoic (20:1 n-9)	0.99	0.45	0.46	0.44
Eicosatrienoic (20:3 n-6)	2.08	2.12	0.20	0.89
Arachidonic (20:4 n-6)	5.02	7.37	0.51	0.01
Eicosapentaenoic (20:5 n-3)	0.36	0.51	0.03	0.02
Bosseopentaenoic (20:5 n-6)	3.01	2.53	0.15	0.06
Docosapentaenoic (22:5 n-6)	20.20	16.53	1.44	0.11
Docosahexaenoic (22:6 n-3)	3.90	4.78	0.32	0.09
Σ SFA	47.79	47.25	0.92	0.69
Σ MUFA	13.36	13.91	1.44	0.79
Σ PUFA	38.66	38.83	1.38	0.93
Σ n-6	34.20	33.53	1.28	0.71
Σ n-3	4.28	5.30	0.32	0.05
n-6/n-3 ratio	8.26	6.41	0.68	0.09


LSM; Least squares means, SFA; Saturated fatty acids, MUFA; Monounsaturated fatty acids, PUFA; Polyunsaturated fatty acid, C; Control, and FO; Fish oil.

## Discussion

The present study assessed the effects of FO supplementation on semen quality and serum testosterone concentrations in dogs. We hypothesized that FO supplementation improved semen quality and increased serum testosterone in dogs. In agreement with our hypothesis, FO supplementation improved their semen characteristics. This finding supported results from previous studies conducted in other species such as pigs (2), turkeys (3) and rams (4), in which FO supplementation improved semen quality. 

This improvement could be due to changes in the sperm phospholipid FA profile (2) due to an increase in n-3 FAs (23). The higher flexibility in the sperm membrane produced by the increase in n-3 FAs might improve the characteristic flagellar motion of sperm (24). Samadian et al. (4) also mentioned that dietary FO supplementation produced changes in the EPA precursors that might be relevant to the alterations in semen quality. In a previous report on dogs by da Rocha et al. (16), daily supplementation with a blend of FAs (n3, omega 6 and omega 9) together with vitamin E, increased sperm concentration, volume and vigor, and decreased morphologically abnormal sperm. In the current study, we found no effects of FO on semen volume or vigor. This discrepancy might be due to the FA blend and the doses of omega and vitamin E used in the former work. In our study, the effect of FO supplementation on semen characteristics could be attributed to the changes in the semen FA profile. Furthermore, the absence of antioxidant supplementation in FO dogs could have influenced the results. Despite the greater number of viable sperm in FO dogs, we observed a higher percentage of viable sperm in C dogs. The decrease in percentage of viable sperm might be due to the lack of extra antioxidants in FO dogs. 

A previous report (10) has stated that the inclusion of vitamin E as an antioxidant may be needed in conjunction with polyunsaturated fatty acids (PUFAs) supplementation in the diet. If antioxidants are not present in an adequate concentration, cellular membrane PUFAs can be oxidized and produce free radicals (25). On the other hand, in our study we have detected a time by treatment effect on total sperm count and total morphologically normal sperm, along with a trend on total sperm viability on day 60 and during the length of the study. The increase in total sperm count might be due to the effect of FO supplementation. According to evidence in rams, FO, as a source of n-3, increased sperm concentration. This increase was due to an acceleration of spermatogenesis. It has been suggested that n-3 FA play an important role on the development of functional sperm (4). Conversely, some studies in boars showed no effects of FO on semen quality or fertility (10). As mentioned above, differences among studies could be due to the species, breed, sources of FA supplements, supplementation period, season, husbandry practices or semen handling procedures (10,26,27). In the current experiment, we evaluated the total FA in semen. FO supplementation increased total n-3 FA in semen samples of FO dogs. The increase in semen EPA concentration might be due to the EPA concentration in FO. During the C and the FO treatment periods, dogs received on average 8.84 mg and 16.72 mg DHA per kg BW0.75, respectively (8.84 mg dietary and 7.88 mg from the supplement). Although FO dogs received almost a double amount of DHA compared to C dogs, there were no significant DHA differences in semen samples in either group, even though we observed a tendency to increase in FO dogs. This non-significant numerical difference might be due to the high variability of DHA in semen or to changes in the efficiency of absorption and utilization of DHA; however, a clear conclusion could not be drawn from the current experiment. Although an unexpected increase occurred in semen AA in FO dogs, we could not explain the physiological mechanism for such an increase, mainly because the AA on the supplement was only 1%. 

Contrary to our expectations, FO supplementation decreased serum testosterone concentrations. To our knowledge, this was the first study which described the effect of supplementation with FO that contained n-3 on serum testosterone in dogs. 

Previous findings in rats (9), humans (28) and boars (10) showed some discrepancies on the effect of n-3 FA supplementation on testosterone concentration. In rats, an association existed between the increase in testosterone concentration after FO supplementation with changes in the number of luteinizing hormone receptors due to the modification of the lipid composition of the Leydig cell membrane (9). Conversely, in humans (28), boars (10) and fish (29), the decrease in testosterone concentrations was related to changes in the synthesis of prostaglandin series 2 to series 3 and the inhibition of prostaglandin formation from AA by EPA/ DHA treatment. On the other hand PUFAs might alter the function of transcription factors that control gene expression such as the peroxisome proliferator activated receptors and affect cellular concentrations of enzymes that regulate both prostaglandin and synthesis of steroids pathways (30). Castellano et al. (10) suggested that variations in the testosterone response to n-3 supplementation might be related to large variations in the type and quantity of n-3 FAs in the diet, to the duration of the supplementation period, and to the animal model used. Based on our results and those from Castellano et al. (10), we assumed that differences could also be due to the total dietary amount of EPA or DHA. In the current experiment, the lack of additional antioxidants in FO dogs could have altered the serum concentration of testosterone due to alterations in the synthesis of steroids (31). 

Further studies are needed to understand the mechanism whereby FO (EPA or DHA) decreases serum testosterone concentration in dogs. Also, there is a need for studies that compare and explain the biology of different responses to FO supplementation among species. 

## Conclusion

Long-term FO supplementation for 120 days might influence semen quality and serum testosterone concentrations in dogs by altering the semen FA profile. 

## References

[1] Stoeckel K, Nielsen LH, Fuhrmann H, Bachmann L (2011). Fatty acid patterns of dog erythrocyte membranes after feeding of a fish-oil based DHA-rich supplement with a base diet low in n-3 fatty acids versus a diet containing added n-3 fatty acids. Acta Vet Scand.

[2] Rooke JA, Shao CC, Speake BK (2001). Effects of feeding tuna oil on the lipid composition of pig spermatozoa and in vitro characteristics of semen. Reproduction.

[3] Blesbois E, Douard V, Germain M, Boniface P, Pellet F (2004). Effects of n-3 polyunsaturated dietary supplementation on the reproductive capacity of male turkeys. Theriogenology.

[4] Samadian F, Towhidi A, Rezayazdi K, Bahreini M (2010). Effects of dietary n-3 fatty acids on characteristics and lipid composition of ovine sperm. Animal.

[5] Castellano CA, Audet I, Bailey JL, Chouinard PY, Laforest JP, Matte JJ (2010). Effect of dietary n-3 fatty acids (fish oils) on boar reproduction and semen quality. J Anim Sci.

[6] Yeste M, Barrera X, Coll D, Bonet S (2011). The effects on boar sperm quality of dietary supplementation with omega-3 polyunsaturated fatty acids differ among porcine breeds. Theriogenology.

[7] Zaniboni L, Cerolini S (2009). Liquid storage of turkey semen: changes in quality parameters, lipid composition and susceptibility to induced in vitro peroxidation in control, n-3 fatty acids and alpha-tocopherol rich spermatozoa. Anim Reprod Sci.

[8] Walker WH (2010). Non-classical actions of testosterone and spermatogenesis. Philos Trans R Soc Lond B Biol Sci.

[9] Sebokova E, Garg ML, Wierzbicki A, Thomson ABR, Clandinin MT (1990). Alteration of the lipid composition of rat testicular plasma membranes by dietary (n-3) fatty acids changes the responsiveness of Leydig cells and testosterone synthesis. J Nutr.

[10] Castellano CA, Audet I, Laforest JP, Matte JJ, Suh M (2011). Fish oil diets alter the phospholipid balance, fatty acid composition, and steroid hormone concentrations in testes of adult pigs. Theriogenology.

[11] Burkholder WJ (2000). Use of body condition scores in clinical assessment of the provision of optimal nutrition. J Am Vet Med Assoc.

[12] Johnston SD (1991). Performing a complete canine semen evaluation in a small animal hospital. Vet Clin North Am Small An Pract.

[13] Soares JM, Avelar GF, França LR (2009). The seminiferous epithelium cycle and its duration in different breeds of dog (Canis familiaris). J Anat.

[14] Cao J, Schwichtenberg KA, Hanson NQ, Tsai MY (2006). Incorporation and Clearance of Omega-3 Fatty Acids in Erythrocyte Membranes and Plasma Phospholipids. Clin Chem.

[15] Nutrient requirements and dietary nutrient concentrations (2006). In: National Research Council.Nutrient requirements of dogs and cats.

[16] da Rocha AA, da Cunha IC, Ederli BB, Albernaz AP, Quirino CR (2009). Effect of daily food suplementation whit essential fatty acids on canine semen quality. Reprod Domest Anim.

[17] Mota Filho AC, Silva HV, Nunes TG, de Souza MB, de Freitas LA, de Araújo AA (2014). Cryopreservation of canine epididymal sperm using ACP-106c and TRIS. Cryobiology.

[18] García Romero G, Tórtora M, Diaz J, Rodríguez R, Abeyá M, Gobello C (2011). A single administration of a GnRH antagonist inhibited canine gonadal axis functionality during 14 days; Milwaukee, Wisconsin, USA.Society for Theriogenology Annual Conference.

[19] Association of Official Analytical Chemists (AOAC) (1990). Official Methods of Analysis.

[20] Folch J, Lees M, Sloane-Stanley GH (1957). A simple method for the isolation and purification of total lipids from animal tissues. J Biol Chem.

[21] Berndtson WE (1991). A simple, rapid and reliable method for selecting or assessing the number of replicates for animal experiments. J Anim Sci.

[22] Richardson CR, Nunnery GA, Wester DB, Cole NA, Galyean ML (2004). Power of test considerations for beef cattle experiments: a review. J Anim Sci.

[23] Gürler H, Calisici O, Calisici D, Bollwein H (2015). Effects of feeding omega-3 fatty acids composition and quality of bovine sperm and on antioxidative capacity of bovine seminal plasma. Anim Reprod Sci.

[24] Strzeżek J, Fraser L, Kuklińska M, Dziekońska A, Lecewicz M (2004). Effects of dietary supplementation with polyunsaturated fatty acids and antioxidants on biochemical characteristics of boar semen. Reprod Biol.

[25] Wander RC, Hall JA, Gradin JL, Du S, Jewell DE (1997). The ratio of dietary (n-6) to (n-3) fatty acids influences immune system function, eicosanoid metabolism, lipid peroxidation and vitamin E status in aged dogs. J Nutr.

[26] Paulenz H, Taugbøl O, Hofmo PO, Saarem K (1995). A preliminary study on the effect of dietary supplementation with cod liver oil on the polyunsaturated fatty acid composition of boar semen. Vet Res Commun.

[27] Estienne MJ, Harper AF, Crawford RJ (2008). Dietary supplementation with a source of omega-3 fatty acids increases sperm number and the duration of ejaculation in boars. Theriogenology.

[28] Nagata C, Takatsuka N, Kawakami N, Shimizu H (2000). Relationships between types of fat consumed and serum estrogen and androgen concentrations in Japanese men. Nutr Cancer.

[29] Wade MG, Van der Kraak G, Gerrits MF, Ballantyne JS (1994). Release and steroidogenic actions of polyunsaturated fatty acids in the goldfish testis. Biol Reprod.

[30] Wathes DC, Abayasekara DR, Aitken RJ (2007). Polyunsaturated fatty acids in male and female reproduction. Biol Reprod.

[31] Chen H, Liu J, Luo L, Baig MU, Kim JM, Zirkin BR (2005). Vitamin E aging and Leydig cell steroidogenesis. Exp Gerontol.

